# Frequency of Missed Findings on Chest Radiographs (CXRs) in an International, Multicenter Study: Application of AI to Reduce Missed Findings

**DOI:** 10.3390/diagnostics12102382

**Published:** 2022-09-30

**Authors:** Parisa Kaviani, Mannudeep K. Kalra, Subba R. Digumarthy, Reya V. Gupta, Giridhar Dasegowda, Ammar Jagirdar, Salil Gupta, Preetham Putha, Vidur Mahajan, Bhargava Reddy, Vasanth K. Venugopal, Manoj Tadepalli, Bernardo C. Bizzo, Keith J. Dreyer

**Affiliations:** 1 Department of Radiology, Massachusetts General Hospital and Harvard Medical School, Boston, MA 02114, USA; 2 MGH & BWH Center for Clinical Data Science, Boston, MA 02114, USA; 3 Qure.ai, Mumbai 400063, India; 4 CARPL, New Delhi 110016, India

**Keywords:** chest X-ray, missed finding, radiology, chest X-ray interpretation

## Abstract

Background: Missed findings in chest X-ray interpretation are common and can have serious consequences. Methods: Our study included 2407 chest radiographs (CXRs) acquired at three Indian and five US sites. To identify CXRs reported as normal, we used a proprietary radiology report search engine based on natural language processing (mPower, Nuance). Two thoracic radiologists reviewed all CXRs and recorded the presence and clinical significance of abnormal findings on a 5-point scale (1—not important; 5—critical importance). All CXRs were processed with the AI model (Qure.ai) and outputs were recorded for the presence of findings. Data were analyzed to obtain area under the ROC curve (AUC). Results: Of 410 CXRs (410/2407, 18.9%) with unreported/missed findings, 312 (312/410, 76.1%) findings were clinically important: pulmonary nodules (*n* = 157), consolidation (60), linear opacities (37), mediastinal widening (21), hilar enlargement (17), pleural effusions (11), rib fractures (6) and pneumothoraces (3). AI detected 69 missed findings (69/131, 53%) with an AUC of up to 0.935. The AI model was generalizable across different sites, geographic locations, patient genders and age groups. Conclusion: A substantial number of important CXR findings are missed; the AI model can help to identify and reduce the frequency of important missed findings in a generalizable manner.

## 1. Introduction

Chest radiography (CXR) is the most performed imaging test, with substantial applications in the screening, diagnosis and monitoring of a variety of cardiothoracic disorders [1,2]. According to some estimates, CXRs represent up to 20% of all imaging exams [3]. Data from the year 2010 reported 183 million radiographic examinations in the United States alone -, with CXRs representing up to 44% of all radiographs [4]. Easy and rapid access, familiarity, low cost and interpretation access all contribute to the widespread use of CXRs.

Despite its overwhelming use, CXR interpretation is subjective and prone to wide interobserver inconsistencies based on readers’ knowledge and experience [5,6,7]. The discordance between radiologists and physicians in one prospective study was 12.5% for CXRs reported as “normal” by physicians but abnormal in the opinion of radiologists [6]. There are also substantial variations among radiologists, with a misinterpretation rate for CXRs as high as 30% in a prior study [8,9]. Not all missed findings are clinically important, but some missed CXR findings have serious implications. Indeed, 19% of early lung cancers that present as nodules on CXRs are missed [10].

To aid the interpretation of CXRs and other imaging modalities, several commercial and research computer programs have been developed and introduced to clinical practice, including those based on artificial intelligence (AI). The AI algorithms can identify patterns and perform complex computational operations more rapidly and precisely than humans [11]. Several studies have reported improved sensitivity, accuracy and efficiency with the use of AI algorithms for the interpretation of CXRs [12,13]. In CXRs, there is a wide range of analyzable findings, with AI algorithms from a single finding (e.g., pneumothorax, lung nodules and pneumonia) to as many as 124 radiographic findings.

We hypothesized that an AI algorithm can reduce missed findings on CXRs. If successful, AI algorithms could help to improve the quality of radiology reports, enhance patient care and help avoid malpractice lawsuits from missed radiologic findings. Although there are multiple prior publications on AI performance, to our best knowledge there are sparse data on the performance of AI algorithms on missed radiological findings. To test the hypothesis, we compared the standalone performance of an artificial intelligence (AI) algorithm for identifying “missed” findings on chest radiographs (CXRs) clinically reported as “normal” against the ground truth according to thoracic radiologists.

### Related Work

Previous studies reported on a considerable frequency of missed findings in chest radiography [14,15]. Hwang et al. reported that AI detected 13.3% of false-negative CXRs in a dataset of 4208 CXRs [16]. Another study by Ahn et al. reported a significant improvement in the detection of CXR findings with an AI algorithm compared to unaided interpretation for all six trained radiologists or trainees [17]. Tam et al. also reported the improved detection of suspicious pulmonary nodules on CXR with AI-aided interpretation (sensitivities 89–94%) versus unaided reporting interpretation for all three radiologists (sensitivities 69–86%), with a slight increase in false positives and a decrease in specificity [18]. Another CXR study reported that standalone AI performance for pneumothorax, pleural effusion and lung lesions was similar to that for radiology residents, but was significantly better than the performance of non-radiology residents [19]. Beyond CXRs, other studies have reported on missed findings of intracranial hemorrhage in noncontract head CT examinations and mammography [20].

## 2. Materials and Methods

### 2.1. Approval and Disclosure

The Human Research Committee of our Institutional Review Board approved the study. The need for written informed consent was waived. Two coauthors (MKK: Coreline Soft Inc., Seoul, South Korea; Riverain Tech., Miamisburg, OH, USA; Siemens Healthineers, Erlangen, Germany; SRD: Lunit Inc., Seoul, Korea; Qure.ai, Mumbai, India) received industrial research grants for unrelated research. AG, PP, BR and MT are employees of Qure.ai, who helped to organize the processing of CXRs but did not take part in case or site selection, ground-truthing or data analysis. SG, VM and VV are employees of Caring Inc. Other coauthors have no pertinent disclosures.

### 2.2. Patients

**Figure 1:**
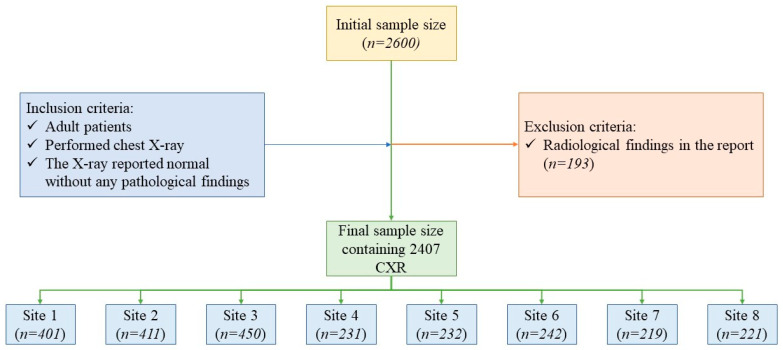
Flow diagram illustrating the patient selection, inclusion and exclusion criteria.

The study data comprised 2407 CXRs from 2407 adult patients (mean age [± standard deviation] 39 [±17] years; male–female ratio 1248:1159) who had a CXR between 2015 and 2021 at one of eight healthcare sites in India (3 sites) or the United States (5 sites) (Figure 1).

At the Indian sites, we used a natural-language-processing-based program embedded within the CARPL Platform (CARPL.AI PVT LTD., Delhi, India) to identify radiology reports of consecutive CXRs reported as normal in all sections of reports from three healthcare sites (Defense Colony Hospital, Hauz Khas Hospital and Safdarjung Hospital; all based in Delhi, India).

At the US sites, we used a radiology report database search engine, mPower (Nuance Inc., Burlington, MA, USA; Microsoft Inc., Redmond, WA, USA), to perform a similar search for CXR reports that were interpreted as normal. Among the US sites, there were two quaternary hospitals (Massachusetts General Hospital and Brigham Women’s Hospital; both in Boston MA) and three community hospitals (Cooley Dickinson Hospital, Northampton, MA, USA; Newton-Wellesley Hospital, Newton, MA, USA; Salem Hospital, Salem, MA, USA). At all sites, search filters were set to include CXRs from patients who were 21 years or older.

The data from each site with the radiology reports were exported in tabular form. Next, we excluded all CXRs with identical medical records or examination numbers to avoid sharing any personal health identifying information across the sites. The resulting data were de-identified and populated into a single Microsoft Excel file (Microsoft Inc. (Redmond, WA, USA)). We selected 250 consecutive CXRs from each of the 5 US sites and consecutive 450 CXRs from each of the Indian sites as the initial study size. A study coinvestigator (PK: a second-year post-doctoral fellow in radiology) reviewed all 2600 CXR reports to exclude 163 CXR reports with description of a radiological finding in any section of the radiology reports (main body, findings or impression sections). Thus, our final study sample size was 2407 CXRs (1262 CXRs from India; 1145 CXRs from US) (Figure 1).

### 2.3. Ground-Truthing

DICOM CXRs of 2407 patients were de-identified and exported offline. All CXRs were then uploaded to a secure-server-based CARPL Annotation Platform (from the Centre for Advanced Research in Imaging, Neuroscience, and Genomics (CARING), Delhi, India) for ground-truthing. Two experienced thoracic subspecialty radiologists (SRD: 17 years of experience; MKK: 14 years of experience) independently reviewed all CXRs on the CARPL platform. Each radiologist commented on the presence of any of the following CXR findings: pleural effusion, pneumothorax, consolidation, lung nodule, opacity (linear scarring or atelectasis), enlarged cardiac silhouette, mediastinal widening, hilar enlargement and rib fracture. We limited the evaluation to these findings because they represented the key detectable findings for the assessed AI algorithm (Qure.ai, Mumbai, India) on CXRs. Since these findings were not reported during clinical interpretation, they were labeled as missed findings.

**Figure 2:**
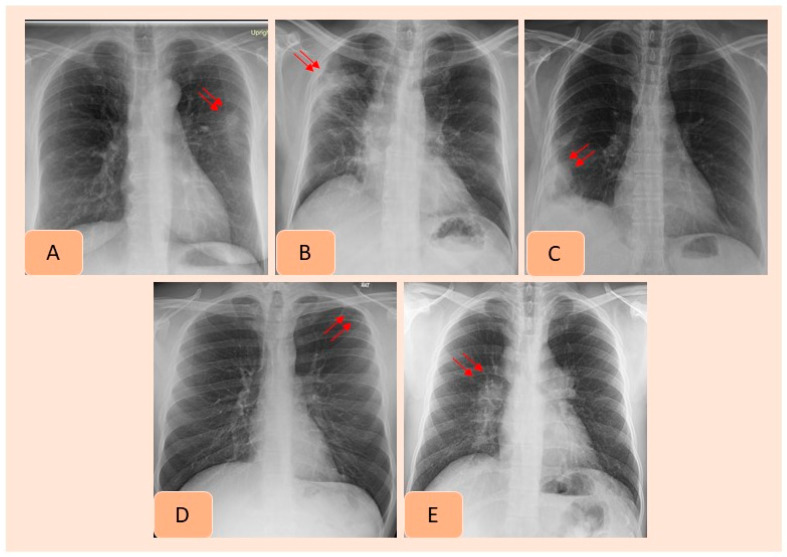
AI-detected CXR findings that were not documented in the radiology reports included pulmonary nodule (**A**), consolidation (**B**), pleural effusion (**C**), pneumothorax (**D**) and hilar prominence (**E**).

For each missed finding, the two radiologists also drew an annotation box within the CARPL Platform (Figure 2) around the finding and gave a score for the perceived clinical importance of the missed finding (1: not clinically important; 2: unlikely of clinical importance; 3: borderline clinical importance; 4: moderate clinical importance; 5: critically important finding). Disagreements between the two radiologists were resolved in a consensus, joint review to establish the final ground truth.

### 2.4. AI Algorithm

All 2407 deidentified frontal CXRs were processed with the AI algorithm (Qure.ai). The ground-truth radiologists had no access to AI output at the time of interpretation. To avoid data sharing and maintain data privacy, all AI processing was conducted behind the institutional firewall of Massachusetts General Hospital.

All 2407 frontal CXRs were exported as DICOM images and processed with an AI algorithm (Qure.ai, Mumbai, India) installed on a personal computer within our institutional firewall. The AI algorithm is cleared for clinical use in 50 countries, including India, but did not have clearance from the US Food and Drug Administration at the time of preparation of this manuscript. The algorithm is based on several convolutional neural networks (CNNs) which identify individual radiographic findings. The specific information pertaining to training and testing of the algorithm has been described in prior studies [21].

Following post-processing of the test datasets, the AI algorithm generated an Excel file with information on model outputs for specific CXR findings based on the probability scores from zero to one hundred. The algorithm also provided a heat map to mark the detected findings on CXRs. The AI outputs were imported into the CARPL platform for data analysis and visualization.

### 2.5. Statistical Analysis

The ground truths and AI output files were uploaded to the CARPL platform for analysis of different radiographic findings based on country, site, finding threshold (vendor-recommended and Youden’s-Index-based), as well as patient gender and age.

We obtained the confusion matrices and area under the receiver operating characteristic (ROC) curve (AUC) from the embedded analytical and statistical functions provided within the CARPL platform. The platform was assessed in a prior research study [22]. In addition, the platform provided an interactive scatter plot to identify the distribution of false-positive and false-negative findings. The findings and country-specific accuracies were calculated based on the vendor-suggested optimal thresholds for individual findings as well as the best performance threshold determination estimated from Youden’s Index with SPSS Statistical Software (SPSS Version 32, IBM Inc., Armonk, NY, USA).

## 3. Results

### 3.1. Prevalence of Missed Findings

**Figure 3:**
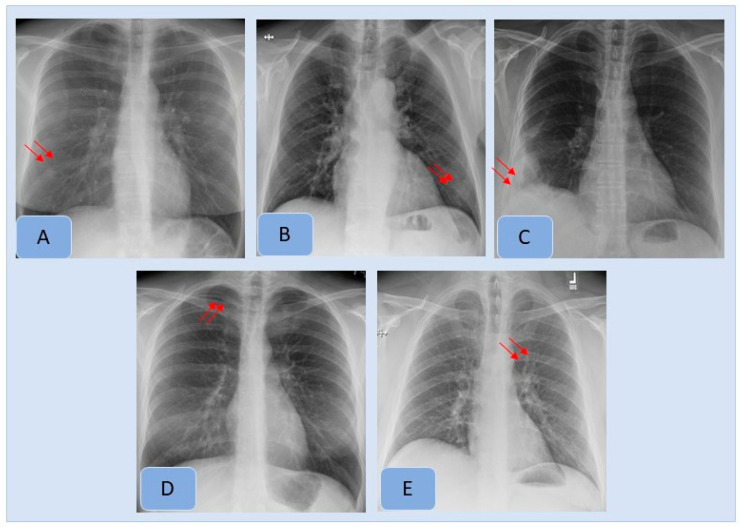
Examples of clinically important missed findings on CXRs included in our study. These included pulmonary nodule (**A**), consolidation (**B**), pleural effusion (**C**), pneumothorax (**D**) and hilar prominence (**E**).

With the ground truth, there were 410 CXRs (17.1%, 410/2407), with missed findings in 342/2407 CXRs (14.2% missed finding rate). The most frequent missed findings included lung nodules (*n*= 177/410, 43.1%), subsegmental atelectasis or scarring (*n =* 67/410, 16.3%), consolidation (*n =* 62/410, 15.1%), enlarged cardiac silhouette (*n =* 35/410, 8.5%), mediastinal widening (*n =* 24/410, 5.8%), hilar enlargement (*n =* 19/410, 4.6%), rib fractures (*n =* 11/410, 2.7%), pleural effusions (*n =* 11/410, 2.7%) and pneumothorax (*n =* 4/410, 0.1%). Figure 3 presents examples of missed findings on CXRs.

Table 1 and Table 2 summarize the distribution of findings without clinical importance (scores 1 and 2) and those with some clinical importance (scores 3–5). The most frequent missed findings without clinical importance included subsegmental atelectasis or scarring (67/137, 62.6%), calcified lung nodules (19/137, 17.8%) and old rib fractures (11/137, 10.2%). The lung nodules deemed as “not important” likely represented calcified granulomata. Likewise, mediastinal widening with little or no clinical importance was related to unfolded thoracic aorta. The most frequent clinically important missed findings included lung nodules (158/273, 52.1%), pulmonary nodules (60/273, 19.8%) and old rib fractures (11/107, 10.3%). Although missed lung nodules were the most frequent missed findings at all sites, the frequency of missed findings varied substantially across the participating sites from India and the US, as well as within each country (*p* < 0001).

### 3.2. Performance of AI Algorithm

**Figure 4:**
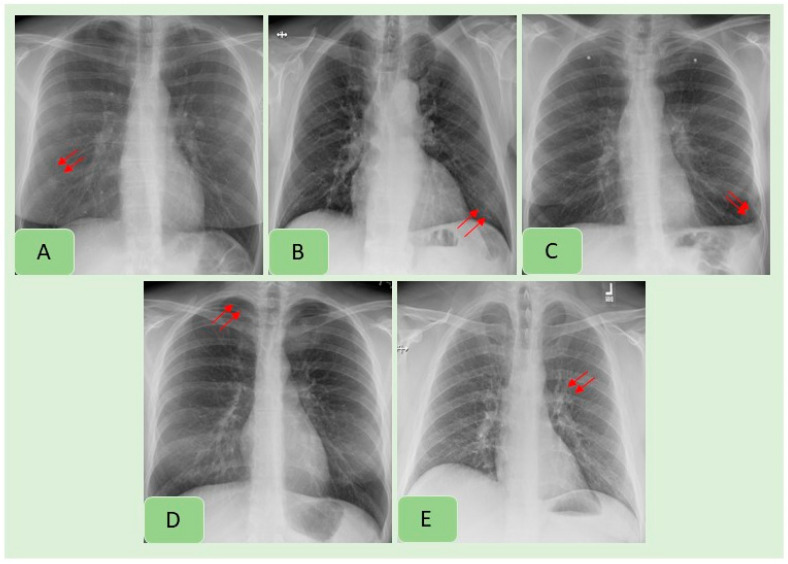
Examples of CXR findings missed by both the AI algorithm and in the original radiology reports: pulmonary nodule (**A**), consolidation (**B**), pleural effusion (**C**), pneumothorax (**D**) and hilar prominence (**E**).

Table 3 summarizes country-wise distribution of CXR findings at the vendor-recommended thresholds. There were variations in the performance of the algorithm across the Indian and US sites, although the differences were not statistically significant (*p >* 0.2). Pneumothorax and mediastinal widening had the lowest AUCs for the AI algorithm, whereas highest AUCs were reported for pleural effusions, enlarged cardiac silhouette, hilar prominence and rib fractures. Figure 2 presents examples of the AI-detected CXR findings which were not reported in the radiology reports. Figure 4 presents findings missed by both the AI algorithm and in the original radiology reports.

**Figure 5:**
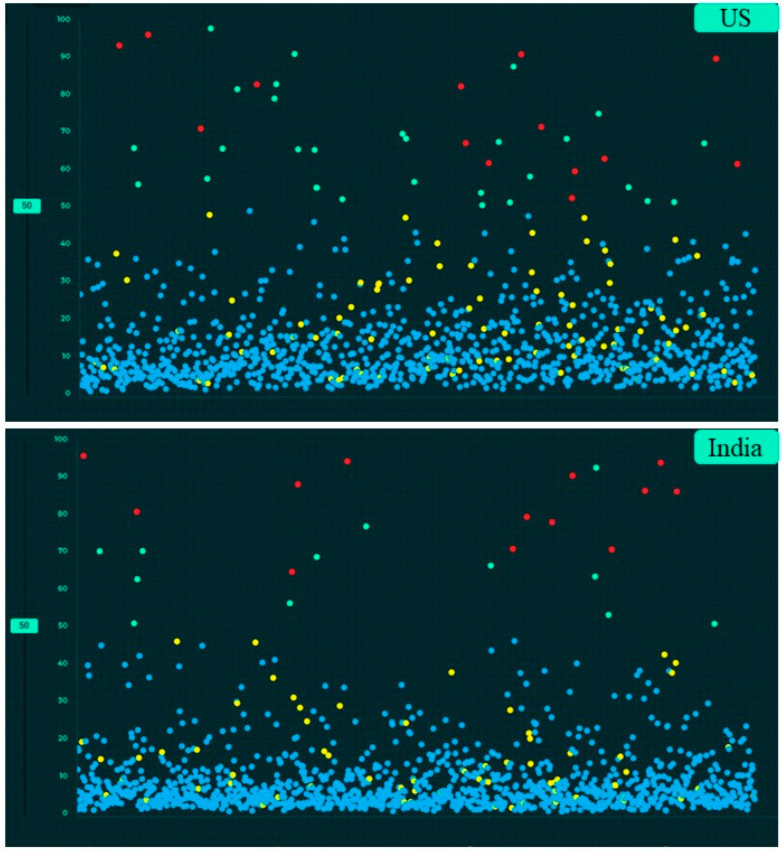
Screen captures of the AI validation platform displaying scatterplots of AI-detected and undetected CXR findings based on country (true positive (red dots), true negative (blue dots), false negative (yellow dots) and false positive (green dots)).

**Figure 6:**
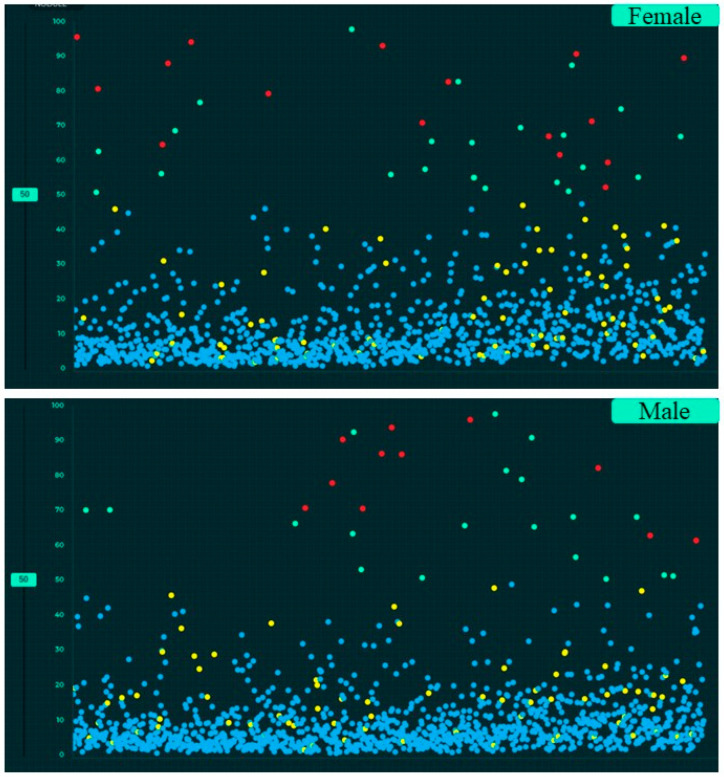
Screen captures of the AI validation interface illustrating the scatterplots of AI output for gender-wise distribution of CXR findings (true positive (red dots), true negative (blue dots), false negative (yellow dots) and false positive (green dots)).

**Figure 7:**
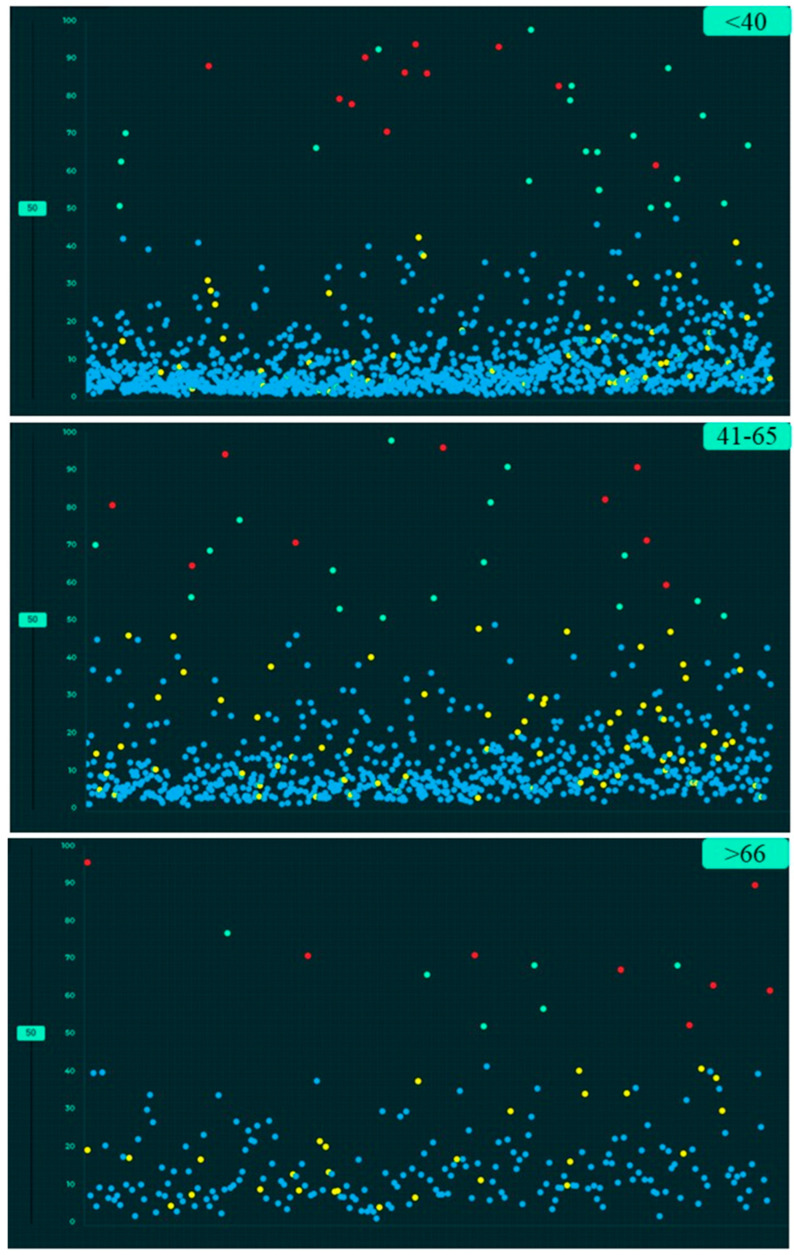
Screen captures of the AI validation platform showing the scatterplots of AI performance based on three age groups for CXR findings (true positive (red dots), true negative (blue dots), false negative (yellow dots) and false positive (green dots)).

Table 4 summarizes the performance of the AI algorithm based on thresholds determined from Youden’s index. There were no significant differences in AI performance based on country or gender (Table 5) (*p* > 0.1). Likewise, there were no significant differences in the performance of the AI algorithm between three different age groups (<40 years, 41–65 years, >65 years) (*p >* 0.05) (Table 6). There were no significant differences in the AUCs for most findings with and without clinical importance (*p >* 0.16). However, the AI algorithm had higher AUC (0.71) for detecting calcified nodules without clinical importance as compared to clinically important, non-calcified pulmonary nodules (AUC 0.55) (*p* = 0.006). Figure 5, Figure 6 and Figure 7 display scatterplots of detected and missed CXR findings with the AI algorithm based on country (Figure 5), gender (Figure 6) and age group (Figure 7).

## 4. Discussion

Our study demonstrates that a substantial number of clinically important findings are missed on CXRs, regardless of practice type and location. The most frequent and clinically important missed findings included lung nodules and consolidation at all eight participating sites in both India and the US. A high frequency of missed lung nodules on CXRs has also been reported in prior studies [23]. Apart from the distribution of missed radiographic findings, our study reports on the performance of an AI validation platform (CARPL) and an AI-CXR algorithm (Qure.ai). The validation platform enabled seamless comparison of AI performance with both summary statistics (e.g., AUCs, accuracies) as well as individual case-level false positives, false negatives, true positives and true negatives. To assess the generalizability of AI results, the validation platform helped to investigate model performance across different findings, participating sites, countries, patient age groups and genders using either vendor-specified or Youden’s-Index-adjusted thresholds.

Although the AUCs for standalone AI performance reported in our study are lower than those in prior studies [24], the assessed AI algorithm detected several missed findings not documented in the original radiology reports. The incremental value of AI for interpreting CXRs in our study follows the trends reported in other AI studies [23,25]. The lower AUCs obtained with the assessed AI algorithm for some missed findings in our study are likely related to the fact that missed findings are more likely to be subtle or difficult to detect, and therefore bring an additional level of complexity to AI performance. Indeed, a recent study from Yen et al. reported that their AI algorithm only detected 19.4% of the unreported lung nodules greater than 6 mm [26]. Likewise, in a real-world dataset of 2972 CXRs, Jones et al. reported that their AI model led to significant changes in report in 3.1% of cases and changes in patient care for 1.4% of patients. The projectional nature of CXRs, the subtlety of radiographic findings and the subjective nature of radiographic interpretation pose similar problems to both AI models and human interpreters. Our study outlines a compelling case for the complementary use of AI in the interpretation of CXRs but stresses the importance of careful primary interpretation of CXRs to avoid missed findings—particularly in patients with lung nodules and consolidation.

Likewise, there are some investigations on pulmonary nodule detection by artificial intelligence in which the system was able to identify more than 99% of the nodules (false positives per image was 0.2) [27]. Furthermore, the AI algorithm could detect fresh, healing and old fractures with high performance (F1-scores, 0.849, 0.856 and 0.770, respectively, with *p* = 0.023 for each) [28].

The chief implication of our study pertains to the validation of AI model performance across multiple sites from two geographically distinct regions of the world. Validation of AI models across diverse datasets is critical for establishing their generalizability. We report on methods and platforms for assessing variations in AI performance based on geographic location, type of hospital setting, patient gender and age group for different types of CXR findings. Users of AI models should be aware of the impact of such variations on their local CXRs. We document the use of an AI validation platform (CARPL) for data annotation and model output analyses of the impact of variables such as age, gender and geographic origin on AI performance. Another implication of our study is the high rate of missed CXR findings at all sites, which is neither a new nor a groundbreaking discovery but stresses the role of AI algorithms in reducing the frequency of such missed findings—at least those deemed clinically important. Although the assessed AI algorithm was not perfect, it successfully detected a substantial number of findings missed by radiologists at eight different sites.

### Limitations 

Our research has some limitations. Several missed findings such as pneumothoraces, pleural effusions and rib fractures were rare (*n* < 11) in our study sample, and therefore it is difficult to assess the performance of the AI model for such findings. Our study limited the number of CXRs per site (250 or 400), whereas a larger number could have yielded a larger number of missed findings—especially for findings with small numbers. Despite a large number of CXRs from 2407 patients from eight sites, including community and quaternary hospitals, the included CXRs primarily originated from two large metropolitan communities. Consequently, the geo-racial variations reported in our study across the US and India could have led to an under- or overestimate of AI performance. However, due to concerns over data privacy and security, multi-site, international studies with thousands of imaging studies are difficult and expensive.

Another limitation of our study is the lack of pediatric CXRs, since the assessed AI model was not trained with adequate pediatric CXRs. Although the assessed AI model could evaluate more than 10 findings included in our study, we did not include other findings due to logistical challenges associated with the interpretation of unfunded studies. Since we assessed the use of only one AI model in our study, we cannot comment on the impact of applying more than one AI model on the overall reduction in missed finding frequency. Future studies should investigate if the use of multiple AI algorithms can further reduce missed finding rates and thereby improve the quality and content of CXR reports. Finally, given the inter-observer variations in radiologists’ interpretation of CXRs, ground-truthing was performed by only two radiologists. However, both radiologists had multiple years of experience as practicing thoracic radiologists and fellowship training in thoracic imaging.

## 5. Conclusions 

Our study shows that the assessed AI algorithm could help to detect a substantial proportion of clinically important missed findings on CXRs. The assessed AI validation platform helped to assess generalizability of AI models across different findings, geographic locations, practice types, patient genders and age groups.

## Figures and Tables

**Table 1:** Summary of site-wise distribution of missed findings (per radiologist ground truth) with “no or likely no” clinical importance, which were not documented in the radiology reports.

Sites	Nodule	Rib Fracture	Pneumothorax	Pleural Effusion	Hilar Enlargement	Mediastinal Widening	Cardiac Silhouette Enlargement	Consolidation	Opacity	All
Site 1	1	5	0	0	1	0	6	0	14	27
Site 2	1	1	0	0	0	0	9	0	10	21
Site 3	2	0	0	0	0	0	3	0	6	11
Site 4	2	1	0	0	0	0	3	0	4	10
Site 5	6	0	0	0	1	1	4	0	9	21
US	12	7	0	0	2	1	25	0	43	90
Site 6	3	0	0	0	0	1	4	0	7	15
Site 7	2	2	0	0	0	1	1	0	6	12
Site 8	2	2	0	0	0	0	5	0	11	20
India	7	4	0	0	0	2	10	0	24	47
All	19	11	0	0	2	3	5	0	67	137

**Table 2:** Summary of site-wise distribution of clinically important missed findings (per radiologist ground truth) in radiology reports which were not documented in the radiology reports.

Site	Nodule	Rib Fracture	Pneumothorax	Pleural Effusion	Hilar Enlargement	Mediastinal Widening	Cardiac Silhouette Enlargement	Consolidation	Opacity	All
Site 1	26	0	1	0	2	2	0	12	0	43
Site 2	18	0	0	0	2	4	0	10	0	34
Site 3	9	0	0	0	2	0	0	11	0	22
Site 4	10	0	0	0	0	0	0	2	0	12
Site 5	24	0	2	3	5	6	0	6	0	46
US	87	0	3	3	11	12	0	41	0	157
Site 6	32	0	1	3	2	3	0	4	0	45
Site 7	27	0	0	4	2	2	0	11	0	46
Site 8	12	0	0	1	2	4	0	6	0	25
India	71	0	1	8	6	9	0	21	0	116
All	158	0	4	11	17	21	0	60	0	273

**Table 3:** Accuracy and area under the curve (AUC) of the AI algorithm based on vendor-based thresholds for different findings on CXRs. The numbers within the parentheses represent 95% confidence intervals.

Findings	India Sites		US Sites	
	Accuracy (%)	AUC (95% CI)	Accuracy (%)	AUC (95% CI)
Opacity	97.93	0.607 (0.472–0.743)	96.06	0.737 (0.661–0.814)
Pleural Effusion	99.52	0.982 (0.955–1.000)	99.65	0.680 (0.325–1.000)
Cardiac silhouette enlargement	99.60	0.977 (0.937–1.000)	97.90	0.916 (0.849–0.983)
Hilar enlargement	99.60	0.786 (0.506–1.000)	98.86	0.642 (0.476–0.808)
Nodule	94.13	0.737 (0.675–0.798)	91.00	0.702 (0.643–0.760)
Rib fracture	99.44	0.789 (0.654–0.923)	99.38	0.86 (0.681–1.000)
Pneumothorax	99.92	0.515 (0.488–0.543)	97.64	0.653 (0.288–0.557)
Mediastinal widening	81.45	0.594 (0.423–0.766)	79.03	0.422 (0.288–0.557)
Consolidation	98.65	0.729 (0.623–0.836)	96.15	0.576 (0.494–0.658)

**Table 4:** Accuracy and area under the curve (AUC) of the AI algorithm based on Youden’s-Index-based thresholds for different findings on CXRs. The numbers within the parentheses represent 95% confidence intervals.

Findings	Accuracy (%)		AUC (95% CI)		Youden’s Index	
	US	India	US	India	US	India
Opacity	92.57	95.87	0.521 (0.430–0.613)	0.520 (0.399–0.640)	0.384	0.307
Pleural Effusion	97.99	98.57	0.666 (0.289–1.000)	0.687 (0.456–0.918)	0.312	0.882
Cardiac silhouette enlargement	93.62	96.27	0.637 (0.507–0.768)	0.800 (0.610–0.989)	0.76	0.896
Hilar enlargement	91.79	96.19	0.500 (0.342–0.658)	0.583 (0.325–0.842)	0.231	0.640
Nodule	90.04	93.82	0.553 (0.489–0.517)	0.580 (0.507–0.652)	0.349	0.361
Rib fracture	99.21	98.81	0.571 (0.334–0.808)	0.499 (0.216–0.782)	0.690	0.615
Pneumothorax	90.91	99.04	0.656 (0.282–1.000)	0.500 (0.000–1.000)	0.441	0.515
Mediastinal widening	79.03	81.45	0.438 (0.296–0.580)	0.546 (0.367–0.725)	0.123	0.269
Consolidation	96.15	98.65	0.499 (0.409–0.588)	0.595 (0.455–0.735)	0.193	0.359

**Table 5:** Variations in the AI algorithm’s performance for detecting different radiographic findings based on patients’ stated gender (female versus male patients). Table represents area under the curve with 95% confidence intervals in parentheses.

Gender	Nodule	Rib Fracture	Pneumothorax	Pleural Effusion	Hilar Enlargement	Mediastinal Widening	Cardiac Silhouette Enlargement	Consolidation	Opacity
Female	0.569 (0.503–0.635)	0.667 (0.2689–1.000)	0.494 (0.000–1.000)	0.642 (0.395–0.888)	0.500 (0.329–0.671)	0.500 (0.342–0.658)	0.707 (0.396–602)	0.499 (0.396–0.602)	0.498 (0.392–0.604)
Male	0.561 (0.491–0.630)	0.498 (0.298–0.699)	0.662 (0.286–1.000)	0.750 (0.434–1.000)	0.563 (0.343–0.782)	0.500 (0.329–0.671)	0.635 (0.438–0.659)	0.539 (0.440–0.638)	0.539 (0.440–0.638)

**Table 6:** Variations in the AI algorithm’s performance for detecting different radiographic findings based on age group (female versus male patients). Table represents area under the curve with 95% confidence intervals in parentheses. (Key: NA—not applicable because there was no missed pneumothorax in patients over 65 years.)

Age (Years)	Nodule	Rib Fracture	Pneumothorax	Pleural Effusion	Hilar Enlargement	Mediastinal Widening	Cardiac Silhouette Enlargement	Consolidation	Opacity
<40	0.577 (0.496–0.659)	0.500 (0.173–0.827)	0.745 (0.299–1.000)	0.600 (0.313–0.886)	0.536 (0.374–0.697)	0.500 (0.268–0.732)	0.666 (0.448–0.884)	0.500 (0.399–0.601)	0.518 (0.404–0.631)
41–65	0.554 (0.484–0.623)	0.623 (0.299–0.947)	0.494 (0.098–0.889)	0.750 (0.216–0.784)	0.500 (0.216–0.784)	0.500 (0.320–0.680)	0646 (0.488–0.805)	0.565 (0.434–695)	0516 (0.404–0.628)
>65	0.580 (0.466–0.694)	0.500 (0.214–0.786)	NA	0.745 (0.299–1.000)	0.500 (0.000–1.000)	0.500 (0.296–0.704)	0.768 (0.563–0.973)	0.498 (0.281–0.715)	0.540 (0.354–0.727)

## Data Availability

Data sharing not applicable.
